# Non-age-related gait kinematics and kinetics in the elderly

**DOI:** 10.1186/s12891-022-05577-2

**Published:** 2022-06-29

**Authors:** Yuanhao Liang, Tinghan Xu, Shichen Qi, Xiang Cao, Eric Hiu Kwong Yeung, Yong Hu

**Affiliations:** 1 Department of Physiotherapy, The University of Hong Kong-Shenzhen Hospital, Shenzhen, China; 2 Department of Orthopaedics and Traumatology, The University of Hong Kong-Shenzhen Hospital, Shenzhen, China; 3 Department of Orthopaedics and Traumatology, The University of Hong Kong, Hong Kong, China; 4 Shenzhen Institute of Research and Innovation, The University of Hong Kong-Shenzhen, Shenzhen, China

**Keywords:** Non-age-related gait analysis, Kinematics, Kinetics

## Abstract

**Background:**

The change of gait kinematics and kinetics along aging were reported to indicate age-related gait patterns. However, few studies focus on non-age-related gait analysis. This study aims to explore the non-age-related gait kinematics and kinetics by comparing gait analysis outcomes among the healthy elderly and young subjects.

**Methods:**

Gait analysis at self-paced was conducted on 12 healthy young subjects and 8 healthy elderly subjects. Kinematic and kinetic features of ankle, knee and hip joints were analyzed and compared in two groups. The degree of variation between the young and elderly in each kinematic or kinetic feature was calculated from pattern distance and percentage of significant difference. The k-means clustering and Elbow Method were applied to select and validate non-age-related features. The average waveforms with standard deviation were plotted for the comparison of the results.

**Results:**

A total of five kinematic and five kinetic features were analyzed on ankle, knee and hip joints in healthy young and elderly groups. The degrees of variation in ankle moment, knee angle, hip flexion angle, and hip adduction moment were 0.1074, 0.1593, 0.1407, and 0.1593, respectively. The turning point was where the k value equals two. The clustering centers were 0.1417 and 0.3691, and the two critical values closest to the cutoff were 0.1593 and 0.3037. The average waveforms of the kinematic or kinetic features mentioned above were highly overlapped with a minor standard deviation between the healthy young and elderly but showed larger variations between the healthy and abnormal.

**Conclusions:**

The cluster with a minor degree of variation in kinematic and kinetic features between the young and elderly were identified as non-age-related, including ankle moment, knee angle, hip flexion angle, and hip adduction moment. Non-age-related gait kinematics and kinetics are essential indicators for gait with normal function, which is essential in the evaluation of mobility and functional ability of the elderly, and data fusion of the assistant device.

## Background

The gait analysis of kinematics and kinetics is an important measurement for evaluating mobility quality and functional ability in the elderly [1–3], providing additional quantitative information for clinical treatments and physiotherapy interventions [4–6]. As well, it plays a vital role in investigating biomechanical mechanisms for developing novel assistant devices, including various rehabilitation or assistant robots [7–9]. Some previous studies reported the differences in gait patterns between healthy elderly and young adults at their self-selected speed, and considered them age-related gait features [10–20]. However, there is few study to report the consistent gait features as non-age-related kinematics and kinetics in the healthy gait. These non-age-related features are essential to perform a defined activity for normal function when other features are changed by age.

According to the literature regarding age-related changes, elderly individuals tend to walk at a slower speed, take shorter step lengths and stride lengths, and spend more time on the double support phase [10–13]. It is widely reported that they adopt altered kinematics and kinetics as a compensation strategy due to muscle weakness or actuator impairment [14, 15], including a smaller hip extension angle and larger hip extension moment, lower ankle plantarflexion, and a reducing range of motion (ROM)[16–18]. These age-related changes persist when walking at different speeds [19–21].

Non-age-related features are useful in various aspects in elderly gait study. To facilitate the evaluation of mobility quality and functional ability in the elderly, a normal reference of kinematic and kinetic features in the healthy population should be established. However, the healthy elderly data is not as easily collected as the young, because the variable geriatric diseases, degenerative diseases or disabilities are not rare in senior subjects. If some gait features can be proved without the effect of aging, those non-age-related gait kinematics and kinetics can be collected from healthy young subjects, which is impactful to enlarge the database for baseline reference of healthy elderly. Moreover, during the clinical assessment, it is sometimes difficult for therapists to differentiate whether the changes in gait result from pathological symptoms or aging compensation. With a good understanding of non-age-related features, therapists could make a clear judgment, because these waveforms are in better intragroup and intergroup consistency and able to reflect the essential functional needs for mobility. In this study, we introduced a time series analysis approach to identify non-age-related features. The selected no-age-related features were also compared and evaluated to the elderly with mild walking issues.

## Methods

### Subjects

A total of 8 healthy elderly subjects (male over 60 years old and female over 55 years old) were recruited for this study. The inclusion criteria of the healthy elderly were self-reported healthy with normal daily activity ability and no walking difficulty. The exclusion criteria included a history of neurological, cardiac, pulmonary or musculoskeletal diseases and memory or cognitive problems with mobility impairment [22], as well as joint pain, low back or leg pain within the past 30 days. A total of 12 healthy young adults under 55 years old were recruited after the test of elderly group. They were selected within a range of body weight and height of the elderly group. The exclusion criteria included any history of neurological, cardiac, pulmonary or musculoskeletal diseases. Women with pregnancies were excluded as well. Two elderly adults with mild walking issues were recruited in the abnormal group. One subject was in bilateral knee pain, especially when walking downstairs and the other had right patella fracture surgery 3 years before the test. This study was approved by the Ethics Committee of The University of Hong Kong - Shenzhen Hospital (2021-032). This study was conducted in accordance with the Declaration of Helsinki.

### Motion capture

The motion capture system was Vicon (Oxford, UK) with 12 infrared cameras at 100 Hz and two force plates (AMTI, USA). A total of 39 spherical markers with a diameter of 14 mm were placed on the anatomical joints in accordance with the Plug-in full limb model [23]. Before gait test, he system was calibrated till the world error of all cameras was less than 2 mm. Each subject performed at least one trial of static pose and ten to fifteen trials of self-selected speed barefoot walking at a 10 m even walkway.

### Data processing and gait measurement

Gait data of each subject were preprocessed on Vicon to screen one to three valid trials. Notably, only the trials with two whole feet on the force plates separately during walking were regarded as valid trials. For each valid trial, two representative gait cycles (left gait cycle and right gait cycle) were selected based on the quality of the data. The corresponding marker trajectories and ground reaction force were recorded.

**Fig. 1:**
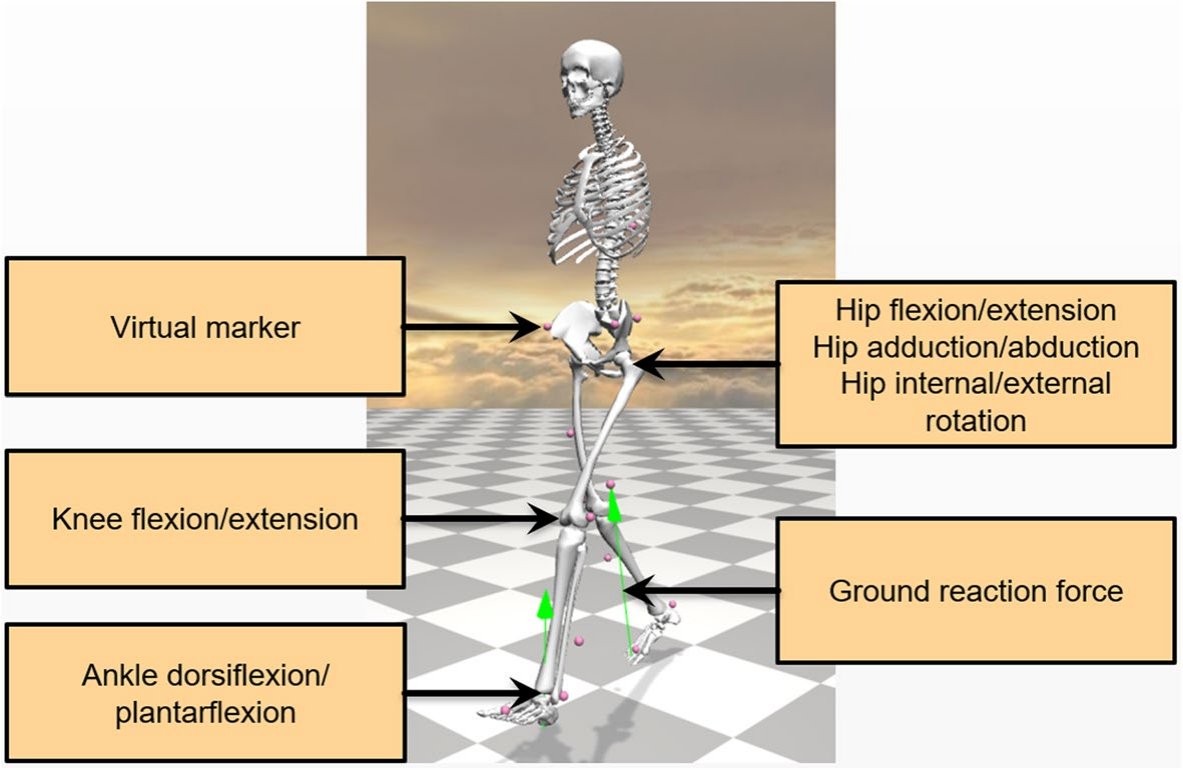
Visualization of Gait2354 model on OpenSim. The joints of interest are noted, including hip, knee and ankle. Pink balls are virtual makers that we set to drive the skeleton. Green arrows denote ground reaction force obtained from the force plates during walking

Opensim 4.1[24] was adopted for the measurement of gait kinematics and kinetics on Gait2354 model [25] (Fig. 1). In this model, the hip joint movement were defined in three degrees of freedom, while movements in knee and ankle joints were defined as one degree of freedom. We attached 16 virtual markers in accordance with the Plug-in lower limb marker placement on this model and additionally added the 10th thoracic vertebrae marker on the trunk. For each subject, the skeleton model was scaled using their static pose to match their anthropometry by minimizing the different locations between the experimental and corresponding virtual markers. We adjusted the location of virtual markers based on the static pose videos to reduce the maximum marker error to less than 4 cm and the root mean square marker error to less than 2 cm. Then an inverse kinematics algorithm [24] with marker trajectories was used to reproduce the gait motion and generate kinematic results of each joint. The inverse dynamics algorithm [24] was applied with ground reaction force to estimate the joint torques. A 1-D interpolation fast Fourier transform method [26] was adopted to normalize the waveforms to the percentage of gait cycle from 0 to 100%. The amplitude of moment-related waveforms was divided by the height and weight of each corresponding subject for the normalization. The final output included ankle angle, ankle moment, knee angle, knee moment, hip flexion angle, hip flexion moment, hip adduction angle, hip adduction moment, hip rotation angle, and hip rotation moment.

### Data Analysis

Independent two-sample t-tests were used to evaluate the amplitude difference between the healthy elderly and young. In each kinematic or kinetic feature, t-test was applied at every time point to examine whether the amplitude showed a significant difference by age(*p* < 0.05). The time percentage with significant differences in each feature was counted. This procedure was named percentage t-test. In addition, the mean value, standard deviation, and ROM of each feature were calculated.

In order to fully consider the temporal information, especially the trend change in the waveform, the pattern distance [27] between the young and elderly was calculated in each feature. The piecewise linear representation was used to divide a waveform into segments along the timeline. The cutoff of each piece was the peak time in the waveform. For each piece, it was marked as 1 with a positive slope, -1 with negative slope, and 0 with no slope. Followed by this, a waveform ${W}_{1}$ with peak number of $i$ could be represented as:

$${W}_{1}=\{\left({s}_{11}, {t}_{11}\right), \left({s}_{12}, {t}_{12}\right),\dots , \left({s}_{1i}, {t}_{1i}\right)\}$$

where ${s}_{1i}\in S=\{1, -1, 0\}$ denotes the slope, and ${t}_{1i}$ are peak times, or the cutoff in other words. For two average waveforms of the young and elderly groups in each feature, piecewise linear representation was applied similarly to the situation of one waveform. To be specific, all peak times in two waveforms were found and ordered. Every waveform was divided into same pieces according to the cutoff. Then the pattern distance of two waveforms ${D}_{{W}_{1}{W}_{2}}$ could be calculated as:

$${D}_{{W}_{1}{W}_{2}}=\sum_{j=1}^{k}{t}_{Nj}*|{s}_{1j}-{s}_{2j}|$$

where $k$ is the total peak number, ${t}_{Nj}=\frac{{t}_{j}}{{t}_{N}}$, ${t}_{j}$ denotes time period of piece j, and ${t}_{N}$ denotes the total time of the waveform. ${D}_{{W}_{1}{W}_{2}}$ was normalized to $[0 , 1]$ by dividing the theoretical maximum value 2.

For each kinematic or kinetic feature, the degree of variation reflected the comprehensive difference of amplitude and temporal change. It was calculated by the mean value of the percentage t-test and pattern distance. Closer to 1 indicates a greater difference. Then k-means clustering algorithm was applied to categorize all the kinematic and kinetic features into two clusters [28]. The non-age-related cluster was those with degrees of variation below 0.2. The changes of the sum of the Euclidean distance with k values were used as the criterion to validate whether the two classes were the most proper to conduct the clustering. To be specific, the turning point of the curve was picked as the number of clusters.

## Results

The healthy elderly group contained four males and four females with an average age of 63.6(SD = 6.14), ranging from 56 to 72 years old. Subjects in this group were measured with a bodyweight of 57.6 (SD = 11.78) kg and a height of 1.63 (SD = 0.10) meters. The healthy young group contained three males and nine females with an average age of 28.7 (SD = 5.17) in a range from 22 to 40 years old. Subjects in this group were measured with a bodyweight of 58.7 (SD = 10.27) kg and a height of 1.66 (SD = 0.07) meters. There was no significant difference in weight and height between the two healthy groups, with *p* values of 0.829 and 0.599 according to the t-test result. The abnormal group included two females aged 71 and 73. They were measured with body weights of 55.9 and 56.3 kg a height of 1.67 and 1.58 m.

The results of data analysis are in Table 1, which is arranged according to the values of degree of variation from the smallest to largest. The clustering results indicate that the first four features are in non-age-related group, including ankle moment, knee angle, hip flexion angle, and hip adduction moment with the degrees of variation of 0.1074, 0.1593, 0.1407, and 0.1593, respectively.

**Table 1:** Results of percentage t-test, pattern distance and degree of variation

Kinematics and Kinetics	percentage t-test	pattern distance	degree of variation	Clustering Results
Ankle Moment	0.1926	**0.2222**	0.1074	Non-age-related
Hip Flexion Angle	0.2593	**0.2222**	0.1407	
Knee Angle	**0.3111**	0.0074	0.1593	
Hip AdductionMoment	0.1778	0.1407	**0.1593**	
Hip Flexion Moment	0.5037	0.1037	**0.3037**	Age-related
Knee Moment	0.5556	**0.0593**	0.3074	
Hip Rotation Moment	**0.4222**	0.2148	0.3185	
Ankle Angle	0.6740	0.1037	0.3889	
Hip Adduction Angle	0.6593	0.1481	0.4037	
Hip Rotation Angle	0.7778	0.2074	0.4926	

This table shows the results of three methods we used in the data analysis. Orders of the results are arranged according to the values of degree of variation from the smallest to largest. The first four lines are non-age-related group based on the k-means clustering results and the rest are age-related group. The bold value denotes the largest and smallest values in the non-age-related and age-related groups respectively

**Fig. 2:**
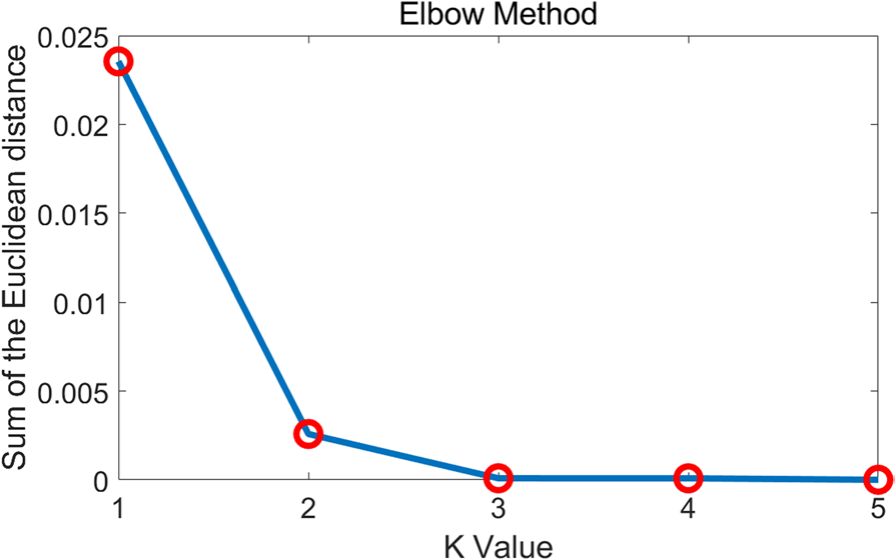
Results of Elbow Method. The horizontal axis is k value we set for the k means clustering and the vertical axis is the corresponding sum of the Euclidean distance. The turning point is where k value equals 2

**Fig. 3:**
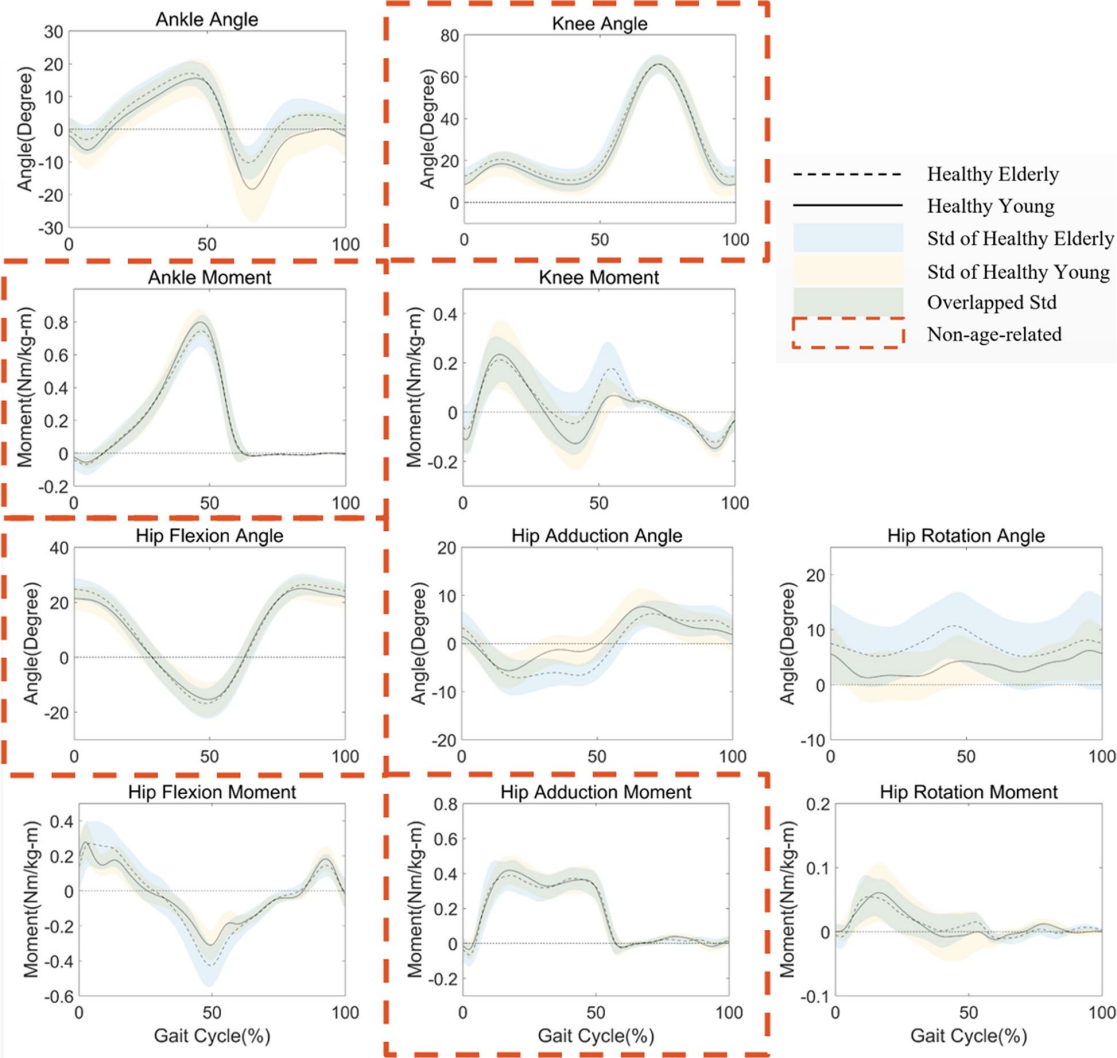
Results of average waveforms with standard deviation for the healthy young and elderly adults, including kinematics and kinetics of interested joints. The horizontal axis is normalized to one gait cycle in percentage from 0 to 100. The black dashed line in each subplot denotes the average waveform of the healthy elderly group and the black solid line denotes the young group. The standard deviation of the healthy elderly group is blue area and that of the young group is in yellow. The overlapped area is in green. Subplots with red dashed contours are determined as the non-age-related group by final k means clustering, which contains ankle moment, knee angle, hip flexion angle and hip adduction moment

**Fig. 4:**
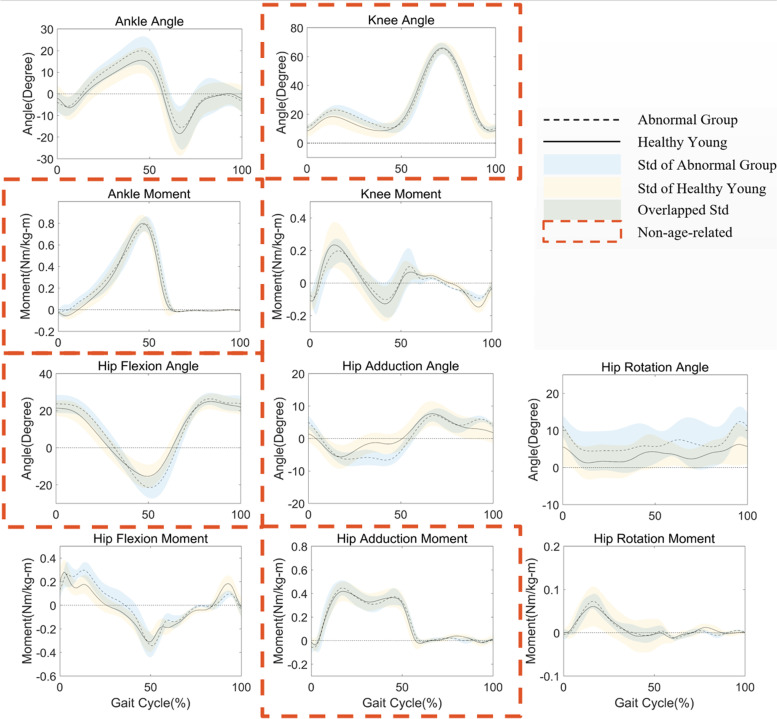
Results of average waveforms with standard deviation for the healthy young and abnormal group. Each subplot is set the same as in Fig. 3. The black dashed line in each subplot denotes the average waveform of the abnormal group and the black solid line denotes the young group. The standard deviation of the abnormal group is blue area and that of the young group is in yellow. The overlapped area is in green. Subplots with red dashed contours are determined as the non-age-related group

The cluster centers of two groups are 0.1417 and 0.3691 and total Euclidean distances are 0.001794 and 0.02750, respectively. The two critical values closest to the cutoff are 0.1593 and 0.3037. Figure 2 shows the turning point is where the k value equals 2, indicating the kinematic and kinetic features are proper to be divided into two groups by k-means clustering. The average waveforms of the healthy with standard deviation are shown in Fig. 3. Four waveforms with red dashed contours are determined as the non-age-related group by clustering. They are highly overlapped with a minor standard deviation between the healthy young and elderly. As a comparison, Fig. 4 shows the average and standard deviation in the abnormal and healthy young. The ROM in kinematics and kinetics for the healthy young and elderly groups is in Table. 2 and the larger values in the healthy elderly group are bolded.

**Table 2:** Range of motion in kinmetics and kinetics for young and elderly group

Kinematics and Kinetics	Young	Elderly
Ankle Angle	36.297 (9.753)	28.701 (5.574)
Knee Angle	62.066 (4.186)	57.707 (5.589)
Hip Flexion Angle	41.432 (5.811)	**44.335 (4.109)**
Hip Adduction Angle	14.626 (3.004)	**15.580 (4.007)**
Hip Rotation Angle	11.652 (4.420)	**14.780 (4.787)**
Ankle Moment	0.878 (0.076)	0.845 (0.084)
Knee Moment	0.458 (0.122)	0.418 (0.083)
Hip Flexion Moment	0.664 (0.140)	**0.780 (0.168)**
Hip Adduction Moment	0.519 (0.099)	**0.540 (0.103)**
Hip Rotation Moment	0.101 (0.035)	0.095 (0.032)

This table shows the average range of motion and corresponding standard deviation of each waveform of kinematics or kinetics. We bold the greater value in the elderly group compared with the young group

## Discussion

Non-age-related kinematics and kinetics are the features in better consistency between the healthy elderly and young adults and are essential to reflect gait in normal function. Due to the lack of neuromusculoskeletal capacity, the elderly tend to adopt compensation strategies with altered muscle recruitment in movement [29, 30], which would lead to some age-related changes in kinematic and kinetic features. Considering this, those unchanged non-age-related features are critical in performing normal gait, because they are the results of compensation. In other words, if the non-age-related features are changed, the normal gait function will get affected. This characteristic could be applied in the clinical as a piece of evidence to differentiate the pathological symptoms from aging compensation. They are also impactful in identifying the representative features and prompt the data fusion of the elderly and young. Mobility devices for the elderly such as exoskeleton robots, assistive robots, and prosthetics, etc., could also be designed similar to the young in these features. Given all these advantages of non-age-related kinematics and kinetics, this study aims to identify these features by comparing normalized waveforms in the elderly and young. Eventually, ankle moment, knee angle, hip flexion angle, and hip adduction moment are determined as non-age-related. This result is supportive of the conclusion in [31]. They utilize the similar gait analysis approach on OpenSim and apply principal component analysis to the kinematic and kinetic features. They point out that sagittal plane joint angles are not different between age groups at the hip or knee, and there were no statistically significant differences between age groups for ankle torque.

The degree of variation calculated from the percentage t-test and pattern distance are adopted to describe the difference between the young and elderly in each feature. The advantages of our analysis methods are shown in the full consideration of the spatial-temporal information and slope changes, and making all the kinematics and kinetics comparable. Some studies do not take into account the temporal information of the waveforms. They directly utilize the maximum and minimum values, and ROM for comparison [17, 32]. Some researchers divide the gait cycle into several pieces based on the key events and phases [33], while this method could not assess the similarity of the waveforms as a whole. In [31, 34], they select principal component analysis to reduce the dimension of gait data and identify the differences between the elderly and young groups. And in [21], they apply t-tests on waveforms and plot h-values along the timeline to indicate the variation. Although these two methods consider the temporal aspect of waveforms, they do not make full use of the amplitude and trend changes over time, which is directly correlated to the interpretation of spatial-temporal gait analysis. In comparison, we use t-test to calculate the percentage that shows significant difference between the young and elderly in each kinematics and kinetics. This method could differentiate amplitude variations very well at every time point. Then the pattern distance along gait cycle axis is calculated to extract the trend difference. This approach compensates the temporal information to percentage t-test and reduces the impact of the absolute amplitude value. Therefore, the kinematics and kinetics are comparable even though the angle values are generally much greater than the moment value. And the degree of variation is introduced for the final classification and validation by k means clustering and Elbow Method.

In the results, the cutoff point of the non-age-related group is very clear. The obvious turning point in Fig. 2 supports that all the features are proper to be clustered into two groups, the non-age-related and age-related. In Table 1, the selected kinematics and kinetics are with close values in each group. We bold the largest value in the no-age-related group and the smallest value in the age-related group. The smallest four values of the percentage t-test are also in the non-age-related group, which is evidence of the same conclusion. However, the two critical values closest to the cutoff (0.3111 and 0.4222) do not show significant variance compared with the degree of variation (0.1593 and 0.3037). In Fig. 3, the waveforms in the non-age-related group show lower intragroup and intergroup variation. The average waveforms are in relatively higher consistency in both amplitude and temporal changes considering the range of motion. And the standard deviation areas are also smaller, and highly overlapped. The hip rotation angle shows the worst performance in both the percentage t-test and degree of variation. We could observe from the waveform that it shows a large deviation in both young and elderly groups and the value of the mean waveform has a significant difference. As a comparison, the ankle moment performs best in the degree of variation. Its intragroup deviation is slight and the mean waveforms are highly overlapped. These results indicate that non-age-related waveforms are consistent with age and individual. In Fig. 4, the non-age-related features show clear variation between the healthy young and abnormal elderly compared with those in Fig. 3, especially knee angle, ankle moment and hip flexion angle. And the age-related features remain large difference. These results prove that non-age-related features are able to reflect abnormal walking function, which is valuable to the clinical evaluation. Moreover, we also find that the values showing larger ROM in the elderly group are all relevant to the hip in Table 2, which could be explained by elderly adults having a greater hip extensor recruitment during gait as a compensation strategy [35].

Although some supportive discoveries in non-age-related gait kinematics and kinetics are revealed by this study, there are also limitations. The results and conclusions are built on the statistical implication of 98 gait cycles from 20 subjects. Future work could involve more data and muscle force for further validation.

## Conclusions

Some kinematic and kinetic features in gait analysis were found identical in healthy young and elderly groups. Ankle moment, knee angle, hip flexion angle and hip adduction moment were identified as the non-age-related features. We find these features are able to reflect normal walking function, which is validated on abnormal elderly. This study result is essential in the evaluation of mobility quality and functional ability of the elderly, and data fusion of the mobility device.

## Data Availability

The data collected and analyzed in the present study are not publicly available due to ethical restrictions but are available from the corresponding author upon request.

## Abbreviation

ROM: Range of motion
